# Agronomic Biofortification of Unconventional Food Plants with Zinc

**DOI:** 10.1021/acsomega.4c06538

**Published:** 2024-11-25

**Authors:** Aline da Silva Costa, Marcelo Henrique Avelar Mendes, Douglas Correa de Souza, Betsy Carolina Muñoz de Páez, Thiago Sampaio Guerra, Paula Aparecida Costa, Paulo Cesar Ossani, Maria Ligia de Souza Silva, Luciane Vilela Resende

**Affiliations:** † Department of Agriculture 545 (DAG), Trevo Rotatório Professor Edmir Sá Santos, Federal University of Lavras (UFLA), Lavras 37203-202, Minas Gerais, Brazil; ‡ Department of Soil Science, Trevo Rotatório Professor Edmir Sá Santos, Federal University of Lavras (UFLA), Lavras 37203-202, Minas Gerais, Brazil; ⊥ School of Agricultural Sciences of Lavras (ESAL), Trevo Rotatório Professor Edmir Sá Santos, Federal University of Lavras (UFLA), Lavras 37203-202, Minas Gerais, Brazil

## Abstract

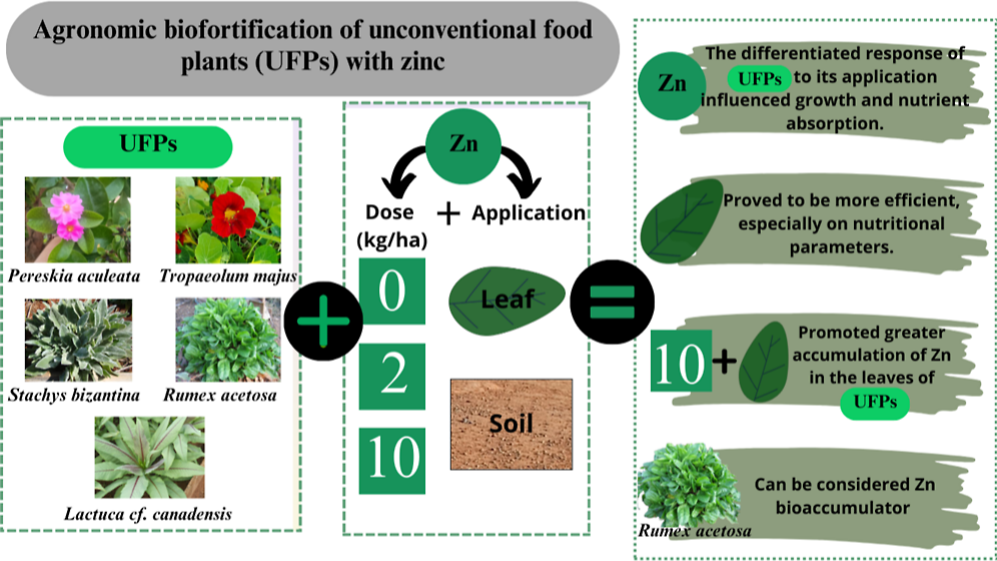

Zinc (Zn) biofortification in food plants presents a good strategy to address inadequate Zn intake by humans, a major health concern. Unconventional food plants (UFPs), known for their rich nutritional profile, offer an accessible and nutritious alternative to the food system. This study evaluated the response of selected UFP species to Zn application. An experiment with a completely randomized design was conducted using a 5 × 3 × 2 factorial scheme with four replicates. Five UFP species: *Lactuca* cf. *canadensis* L (Lc), *Pereskia aculeata* (Pa), *Rumex acetosa* (Ra), *Stachys byzantina* (Sb), and *Tropaeolum majus* (Tm) were tested with three Zn doses (0, 2, and 10 kg ha^–1^) and two application methods (soil and foliar). The parameters evaluated included leaf number, chlorophyll content, fresh and dry mass, moisture, and mineral content. Foliar application proved to be the more efficient method, with Ra and Sb showing the greatest Zn accumulation. Kohonen’s self-organizing maps efficiently explored correlations and groupings, revealing that Zn application influenced these attributes. Biofortified leaves of UFPs show strong potential in mitigating Zn nutritional deficiencies.

## 1 Introduction

Zinc plays a multifaceted role in biological systems,^1^ functioning as an essential micronutrient required in small quantities by humans. It is crucial for processes such as growth and development, immune system function, reproductive health, sensory function, and neurobehavioral development.^3^ This is because zinc is the only metal associated with all enzyme classes; currently, more than 300 enzymes and more than 1000 transcription factors are known to require zinc for their activity.^2,4^

For this reason, zinc deficiency poses significant risks to human health, to the extent that it has been classified as the fifth greatest health risk factor in developing countries and the 11th globally.^5^ The highest-risk populations for Zn deficiency are concentrated in South and Southeast Asia, Sub-Saharan Africa, Central America, and South America-regions where diets are primarily plant-based and the intake of animal-derived foods is low.^6^

In contrast to other metals, such as iron, copper, and mercury, which can accumulate to toxic levels in the body, zinc absorption, subcellular distribution, storage, and excretion are efficiently controlled.^7^ However, excessive zinc intake can cause some short-term side effects such as vomiting, and high zinc intakes relative to copper can induce copper deficiency.^9^ Therefore, caution is necessary when considering zinc supplementation,^10^ with priority given to zinc-rich foods with high bioavailability,^8^ in reasonable quantities.

In this sense, to improve zinc intake through dietary interventions, especially in populations most affected by its deficiency, it is important to consider their existing eating habits, ensuring, whenever possible, zinc intake through these foods. For rural populations, which are the most affected by zinc deficiency, the consumption of unconventional food plants (UFPs) has historically been part of their diet.^11^ UFPs refer to edible plants that generally lack market value or are sold only on a small scale.^12^ These plants, often defined as unknown or underutilized in urban areas,^13^ have gradually been forgotten or devalued due to changes in eating habits. Recovering the use of UFPs is not only vital for preserving biodiversity,^12^ but also for taking advantage of their nutritional benefits.^14,15^

In this context, UFPs are food species that contribute to the sustainability of production and consumption,^11,14,16^ thus aligning with the Sustainable Development Goals (SDGs), especially the goal of achieving zero hunger through sustainable agriculture.^17^ Furthermore, UFPs represent a promising alternative for reallocating and revitalizing local or traditional food systems, improving food security.^11,18,19^ These underutilized and nutrient-rich food resources provide opportunities to diversify diets,^11,18^ especially in areas where food insecurity is prevalent.

Evaluating the ability of these species to absorb and transfer zinc to their edible parts, considering their genetic variability, is an important step to reducing zinc deficiencies through biofortification.^11,16,20,21^ This method is understood as the increase in the nutritional value of crops, offering a solution for vitamin and mineral deficiencies, including zinc.^20,22^ Plant biofortification can be achieved traditionally, via soil or foliar mineral nutrition,^20^ which involves the application of any dissolved mineral nutrient directly to the plant’s foliage.^21^

Several authors have stated that the differences between soil and foliar fertilization lie in the efficiency of nutrient absorption. When applied to the soil, mineral fertilizers depend on their transformation to be absorbed by plants, whereas foliar application allows for the direct use of nutrients by the plant’s metabolic processes.^20,23,24^

Therefore, considering the importance of UFPs as an alternative for dietary diversification, and the need for zinc in human nutrition, this research aimed at assessing the nutritional and productive response of five UFP species to zinc biofortification.

## 2 Materials and Methods

### 2.1 Experimental Location and Conditions

The study was conducted between April and August 2019 in a greenhouse in the Olericulture sector of the Department of Agriculture at the Federal University of Lavras (UFLA), located in Lavras, southern Minas Gerais, Brazil (latitude 21° 14′ S, longitude 45° 00′ W, altitude 918.8 m). According to the Köppen climate classification, the region’s climate is characterized as Cwb mesothermal, with dry winters and rainy summers.^25^

The plant materials used for propagation were obtained from the UFLA unconventional vegetable germplasm collection. The species *Tropaeolum majus* (Tm) and *Lactuca*cf.*canadensis* L. (Lc) were sown in plastic trays with 200 cells filled with commercial substrate (Tropstrato HT Hortaliças, Vida Verde). *Pereskia aculeata* (Pa) was propagated by cuttings from the apical meristem, while *Rumex acetosa* (Ra) and *Stachys byzantina* (Sb) were propagated through tussock shoots.

Once the plants were adequately developed, they were transplanted into plastic pots containing 4 dm^3^ of soil. We used native tropical Cerrado soil (Oxisols), with its chemical and physical characteristics shown in Table 1. Soil acidity was corrected by applying calcium carbonate (PA) to increase the base saturation to 80%. Planting fertilization followed the recommendation of Malavolta.^53^ Nitrogen and potassium were applied in split doses, and all other nutrients were applied in a single dose.

**Table 1:** Soil Chemical and Physical Properties of Native Cerrado Soil (Tropical Soil) Used in the Experiment^a^

pH	Al (cmol_c_ dm^–3^)	H + Al (cmolc dm^–3^)	Ca (cmolc dm^–3^)	Mg (cmolc dm^–3^)	SB (cmolc dm^–3^)	CEC (cmolc dm^–3^)	V (%)	P (mg dm^–3^)	K (mg dm^–3^)	S (mg dm^–3^)
5.8	0.04	1.47	0.76	0.10	0.88	2.35	37.6	0.15	9.18	37.48

Cu (mg dm^–3^)	Fe (mg dm^–3^)	Zn (mg dm^–3^)	Mn (mg dm^–3^)	Clay (g kg^–1^)	Silt (g kg^–1^)	Sand (g kg^–1^)	OM (g kg^–1^)	Texture
2.75	28.70	0,80	8.50	340	300	360	7	clay loam

a pH: soil pH; exchangeable K, Ca, Mg, and Al; H + Al: potential acidity; SB: sum of bases; CEC: cation exchange capacity; V: base saturation, P: available phosphorus (mehlich); S: sulfur; OM: organic matter.

Three doses of zinc (0, 2 and 10 kg ha^–1^) were applied using zinc sulfate as fertilizer. Zinc was applied 22 days after transplanting in a single dose when applied via soil. The fertilizer was diluted in 35 mL of water per pot for leaf applications. In treatments with 0 and 2 kg ha^–1^, the foliar applications were carried out in a single dose, whereas 10 kg ha^–1^ dosage was divided into three equal applications.

Throughout the experiment, the plants were irrigated to a total water depth of 2.5 mm day^–1^ during the three periods. Leaf size at harvest and the number of days after planting (DAP) are summarized in Table 2.

**Table 2:** Leaf Size Requirements for UFP Harvest and Average Days After Planting (DAP) to Achieve It

Specie	Leave size (cm)	DAP
*Tropaeolum majus*	3–10^a^	50
*Lactuca cf. canadensis* L.	20–25^b^	60
*Pereskia aculeata*	>7^b^	60
*Rumex acetosa*	10–15^b^	50
*Stachys byzantina*	10–15^b^	60

a Diameter.

b Long.

### 2.2 Experimental Design

The experiment followed a completely randomized design (CRD) with four replications. A 5 × 3 × 2 factorial scheme was employed, with five leafy UFP species (Tm, Lc, Pa, Ra, and Sb), three levels of zinc (0, 2 and 10 kg ha^–1^), and two application methods (soil and foliar), resulting in a total of 120 pots.

### 2.3 Variables Analyzed

#### 2.3.1 Agronomic Variables

The following evaluations were carried out: the chlorophyll content was determined by the relative chlorophyll index (SPAD index) using a SPAD-502 chlorophyll meter. The analysis was carried out 35 days after transplanting on fully developed leaves of the upper third of the plants. The hue angle (HA) was measured using a Konica Minolta CR-400 colorimeter calibrated according to the CIE system, and L*, a* and b* values (illuminant D65) were recorded close to the harvest time. The height (He) was measured using a ruler from the soil surface to the plant apex; the number of leaves (NL) was determined by performing a single count for each plant; the fresh mass (FM) was measured after harvesting the aerial parts of the plants (leaves and flowers); and the dry mass (DM) and moisture (Moist) were determined after drying the aerial plant parts in a forced-air oven at 65 °C until constant weight was achieved.

#### 2.3.2 Nutritional Variables

Zinc and other minerals (N, P, K, Ca, Mg, S, B, Cu, Fe and Mn) were quantified using the element analysis method for plant material described by Malavolta.^26^ Nitrogen was determined by the semimicro-Kjeldahl method, while the other minerals were measured using inductively coupled plasma optical emission spectrometry (ICP-OES).

### 2.4 Data Analysis

The data were subjected to analysis of variance (ANOVA). When significant differences were found, means were compared using the Scott–Knott test at a 5% significance level. The analyses were performed using R software.^27,28^

### 2.5 Kohonen Self-Organizing Map

In this research, a Kohonen self-organizing map was created to group treatments into clusters based on the similarity of their properties. The SOM Toolbox 2.1 package^29^ in MATLAB R2015a was used, with modifications to improve the acquisition and validation of data clusters, using the Davies–Bouldin and Silhouette indices.

## 3 Results

### 3.1 Analysis of Agronomic Variables

**Figure 1:**
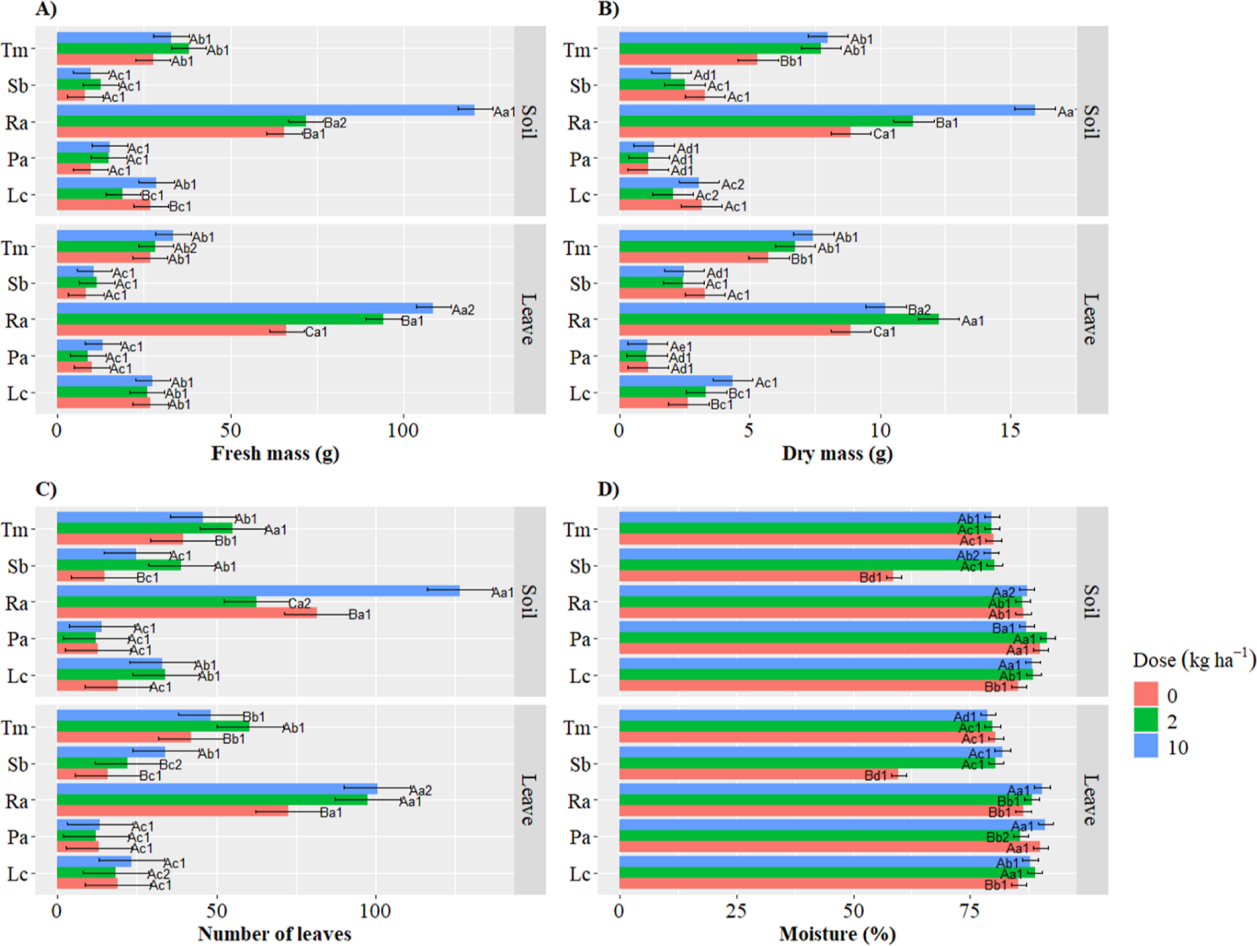
Agronomic variables with significant interaction for species-dose-application method. (A) Fresh mass; (B) dry mass; (C) number of leaves; (D) moisture. Species evaluated: Lc = *Lactuca cf. canadensis* L.; Pa= *Pereskia aculeata*; Ra= *Rumex acetose*; Sb = *Stachys byzantina*; Tm= *Tropaeolum majus.* Different capital letters indicate significant differences between doses within the same species and application method. Different lowercase letters denote significant differences between species within the same dose and application method. Numbers represent significant differences between application methods within the same species and dose. Test: Scott–Knott. *P*-value: 0.05.

Significant interactions (*p* ≤ 0.05) were observed among the three evaluated factors for the agronomic variables fresh mass (FM), dry mass (MS), number of leaves (NL) and moisture content (Moist) (Figure 1).

As shown in Figure 1, the species *R. acetosa* (Ra) presented the greatest variability across the agronomic parameters, except for moisture content, where *Stachys byzantine* (Sb) demonstrated the most significant variation. Thus, Ra showed a progressive increase in FM and NL because of the increasing zinc doses, with the greatest improvements occurring when zinc was applied foliarly. In contrast, *P. aculeata* (Pa) showed no variation in FM, MS, and NL based on different doses or application methods. Similarly, the evaluated factors only influenced NL in Sb, with the highest value occurring at a dose of 2 kg ha^–1^ when the fertilizer was applied to the soil, while foliar application at 10 kg ha^–1^ produced the best result.

For *T. majus* (Tm), soil application of Zn increased FM production by 34.93% at a dose of 2 kg ha^–1^ and 18.20% at 10 kg ha^–1^, compared to the control. In contrast, soil application of 2 kg ha^–1^ zinc to *Lactuca cf. canadensis* (Lc) reduced FM production by 29.90%.

Regarding DM content, no differences were observed in Lc with a 10 kg ha^–1^ soil application, while foliar application led to a higher DM content. Similarly, Tm exhibited higher DM at both 2 and 10 kg ha^–1^, with no significant differences between these doses. Sb and Pa showed no significant differences, regardless of the dose or the application method (Figure 1).

On the other hand, for Sb, foliar application of 10 kg ha^–1^ Zn increased NL by 44.38% (±4.41), while soil application at 2 kg ha^–1^ promoted the greatest increase in NL. Similarly, either foliar or soil application of Zn at 2 kg ha^–1^ increased NL for Tm. On the contrary, soil application of the same dose reduced NL by 23.31% for Ra, while 10 kg ha^–1^ Zn increased NL by 35.57% in this species, compared to the control.

The height (He) variable did not show any significant interactions, although differences were observed among individual factors, such as species and dose (*P* value < 0.05). The effect on the species was as follows, from lowest to highest: Lc = Ra < Sb = Tm < Pa. Among the applied doses, 2 and 10 kg ha^–1^ resulted in equal averages, while the control treatment (0 kg ha^–1^) had a significantly lower mean.

**Figure 2:**
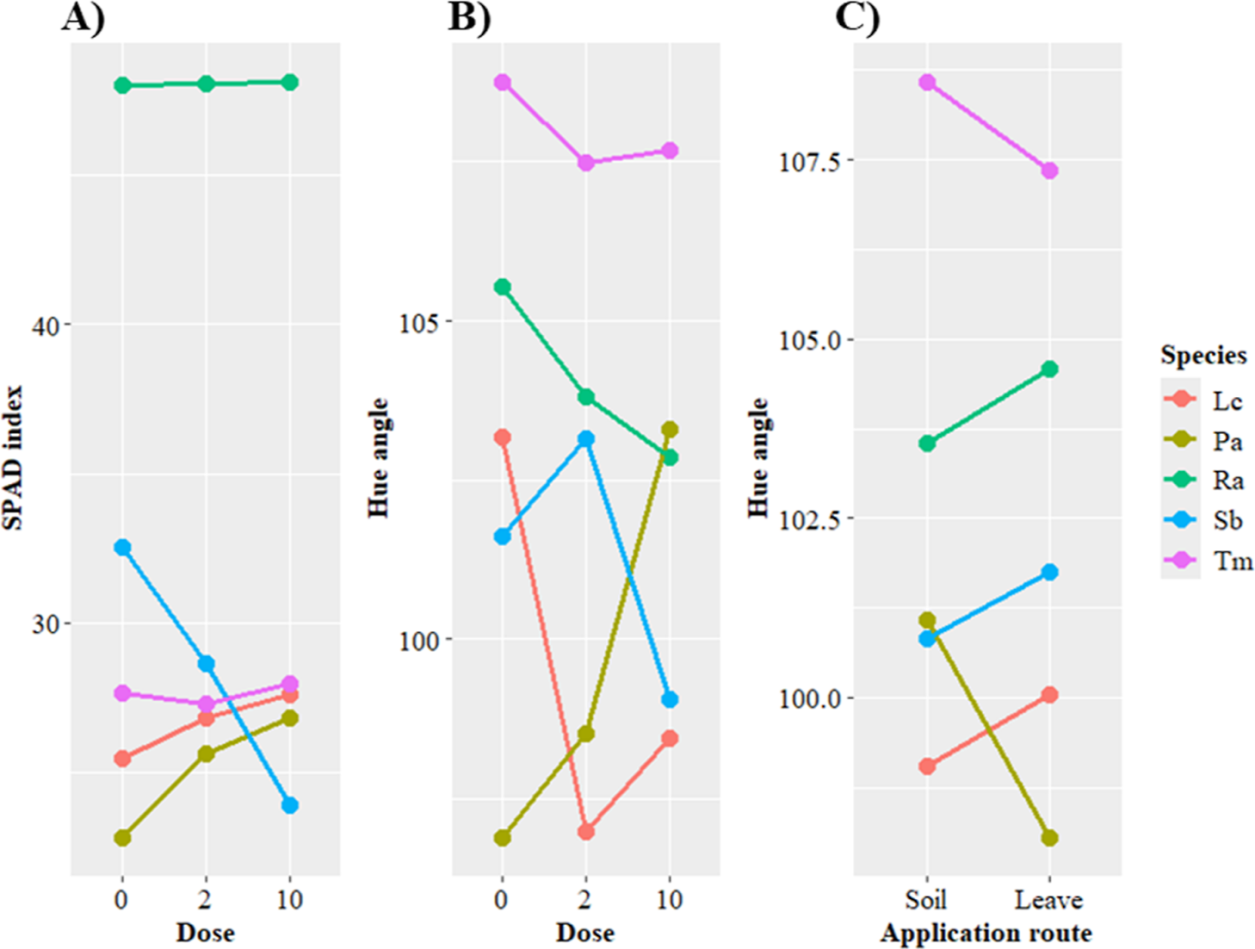
Plots of significant interactions for SPAD index and hue angle. (A) Dose-species interaction based on the SPAD index; (B) dose-species interaction based on the hue angle; (C) application method–species interaction based on the hue angle. Species evaluated: Lc = *Lactuca cf. canadensis* L.; Pa = *Pereskia aculeata*; Ra = *Rumex acetose*; Sb = *Stachys byzantina*; Tm = *Tropaeolum majus*.

For the SPAD index, there was a two-way interaction between the dose and species factors, while for hue angle (AH), a two-way interaction was observed between dose and species, and between application method and species (Figure 2). The dose-species associations demonstrated that Ra showed the highest SPAD index at all doses, including in the control (Figure 2A). For Sb, the SPAD index was inversely proportional to the increase in Zn dose, while Tm presented a complex response to the doses, with the lowest SPAD index at 2 kg ha^–1^ Zn. In addition to simple interactions, the other species evaluated displayed a directly proportional response to the Zn doses (Figure 2A).

Regarding AH values, the dose-species and the application method–species interactions showed that Tm had the highest values. The dose-species interaction (Figure 2A) was complex for Lc, Sb and Tm; that is, there was a change in the AH ranking depending on the dose applied. For Pa, the interaction was simple and directly proportional, while for Ra, the interaction was simple but inversely proportional; that is, an increase in the Zn dose led to a decrease in the AH value. Considering the interaction between species and application method, Lc, Ra and Sb had the highest values when Zn was applied to the soil, while foliar application favored Pa and Tm.

### 3.2 Nutritional Variables

#### 3.2.1 Macroelements

**Figure 3:**
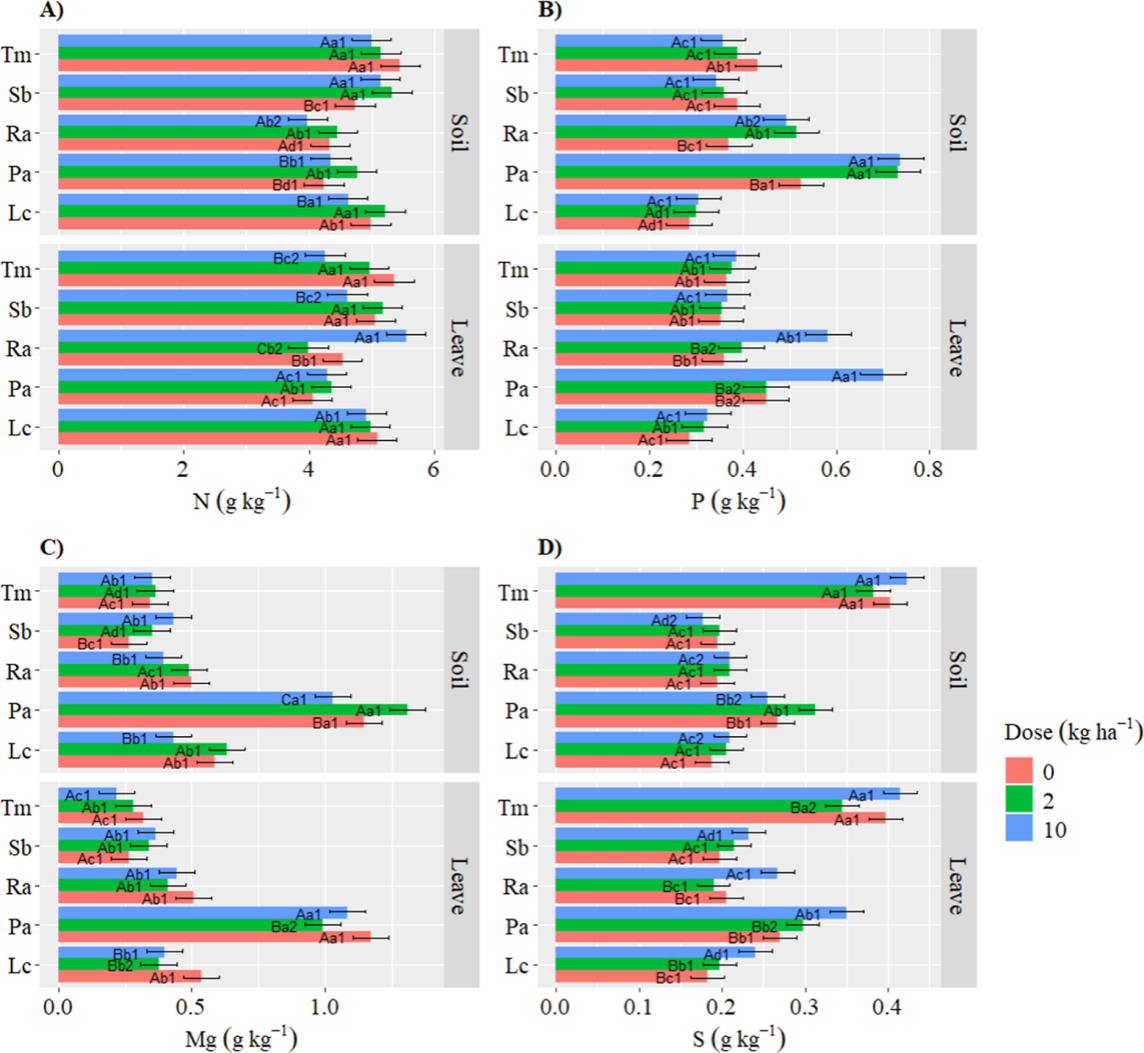
Macroelements with significant species-dose-application method interactions. (A) Nitrogen (N); (B) phosphorus (P); (C) magnesium (Mg); (D) sulfur (S). Different capital letters indicate significant differences between doses within the same species and application method. Different lowercase letters denote significant differences between species within the same dose and application method. Numbers represent significant differences between application methods within the same species and dose. Test: Scott–Knott. *P*-value: 0.05.

Significant differences were observed between the species within each dose and each application method for nitrogen (N) concentration. The highest N content was recorded for Ra (5.54 g kg^–1^) with foliar application of 10 kg ha^–1^ Zn. In addition, soil application of Zn to Tm and Ra and foliar application of Zn to Lc and Pa did not affect N concentration. However, foliar Zn application reduced N content in Sb and Tm, while soil application reduced N levels in Lc (Figure 3).

Both foliar and soil Zn applications favored higher concentrations of phosphorus (P) in the leaves of Pa and Ra (Figure 3B). In the other treatments, no significant differences in the P level were observed.

The impact of Zn on magnesium (Mg) concentrations was more evident in Sb than in other species, regardless of the doses or application methods. For Pa, a 2 kg ha^–1^ Zn dose applied to the soil significantly increased Mg concentration, whereas foliar application at the same dose resulted in significantly lower values than those in the control for this species. Likewise, the soil application of 10 kg ha^–1^ Zn reduced the concentration of Mg in Ra and Lc, regardless of the application method.

Conversely, soil Zn application increased the value of sulfur (S) in Pa only at 2 kg ha^–1^ dose. Foliar application of 10 kg ha^–1^ Zn significant increased S concentration for Lc, Pa and Ra compared to other doses within the same species and between application methods.

**Figure 4:**
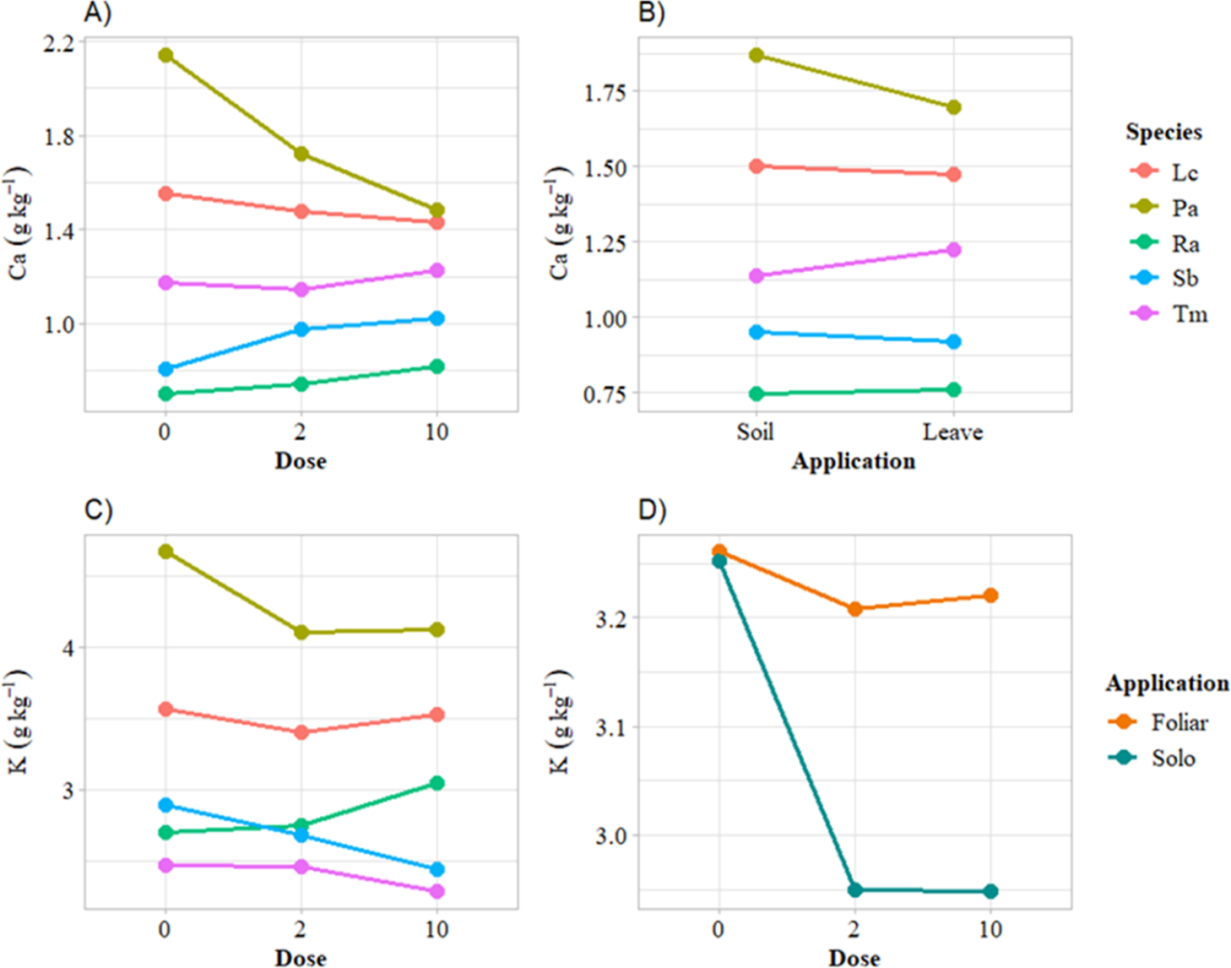
Response of treatments on macroelements calcium (Ca) and potassium (K). Species evaluated: Lc = *Lactuca cf. canadensis* L.; Pa = *Pereskia aculeata*; Ra = *Rumex acetose*; Sb = *Stachys byzantina*; Tm = *Tropaeolummajus*.

For calcium (Ca), the dose-species interaction analysis (Figure 4A) showed that the concentrations in the leaves of two UFPs, namely, Lc and Pa, decreased with increasing Zn dose. On the other hand, Ca concentrations in Ra and Sb significantly increased at doses of 2 and 10 kg ha^–1^. In Tm, Ca concentrations decreased at 2 kg ha^–1^. In terms of the application method–species interaction, Ca values were significantly greater in Lc, Pa, and Sb when Zn was applied to the soil, while foliar application favored significantly greater concentrations in Ra and Tm.

The behavior of potassium (K) was similar to that of Ca in the dose-species interaction (Figure 4C), except for Sb, where K concentration decreased as Zn doses increased. Considering the interaction between dose and application method (Figure 4D), soil application of Zn consistently produced a decrease in K values, regardless of the dose applied.

#### 3.2.2 Microelements

**Figure 5:**
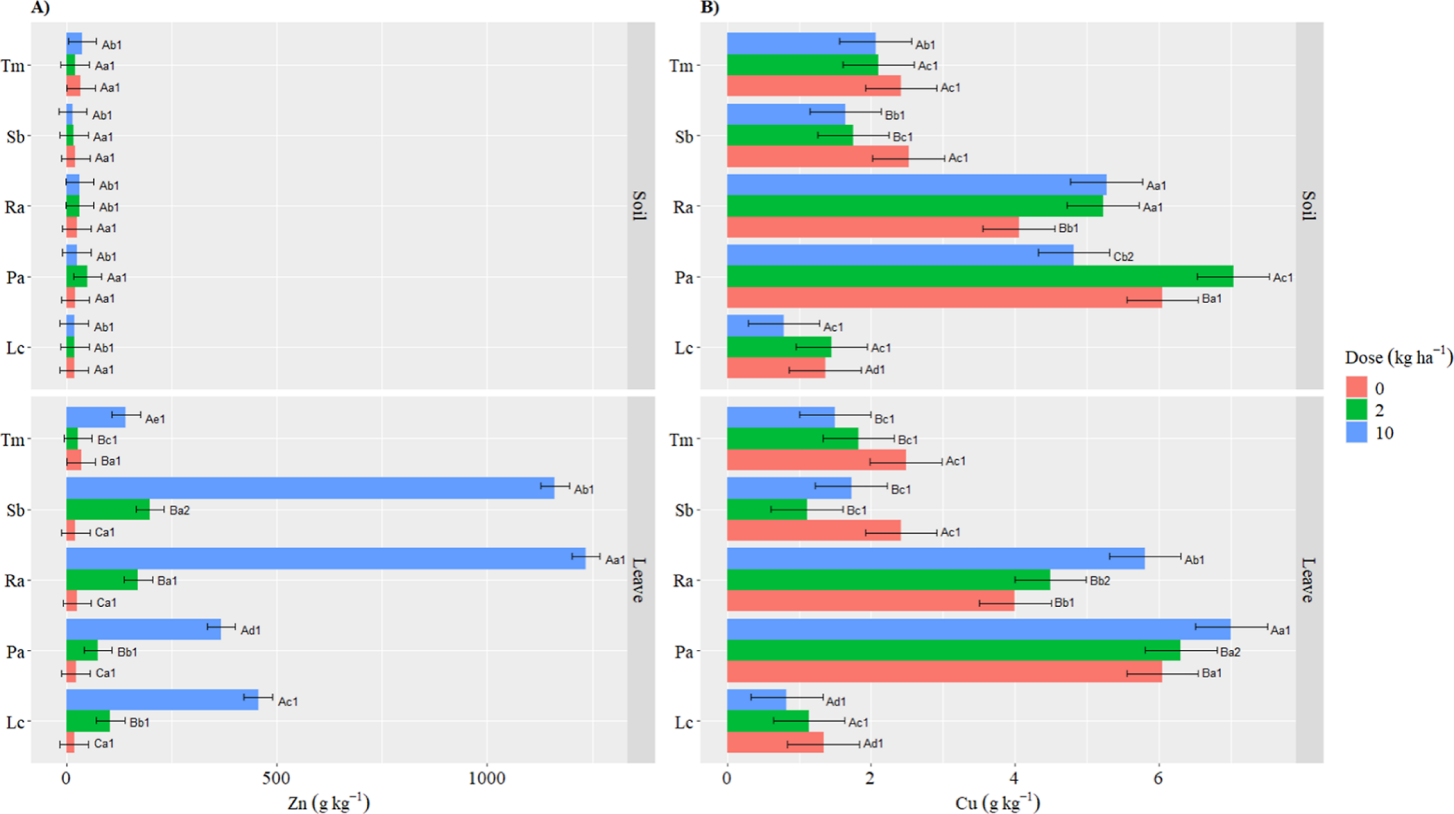
Microelements with significant interaction for species—dose-application method. Zinc (Zn) and copper (Cu). Different capital letters indicate significant differences between doses within the same species and application route. Lowercase letters indicate significant differences between species within the same dose and application method. Numbers represent differences between application methods within the same species and dose. Test: Scott–Knott. *P* value: 0.05.

The application of Zn to the soil did not significantly increase Zn concentration in plant tissues (Figure 5A); however, when 10 kg ha^–1^ of Zn was applied to the leaves, Zn concentration increased drastically: by 400% in Tm, 1600% in Pa, 2430% in Lc, 4840% in Ra, and 5320% in Sb, compared to the control. The application of Zn led to a significant decrease in the copper (Cu) concentration in Sb and Tm, regardless of the application method. In contrast, Pa had significantly higher Cu values when 2 or 10 kg ha^–1^ Zn was applied foliarly. In Ra, Cu concentrations were high regardless of the application method (Figure 5B).

**Figure 6:**
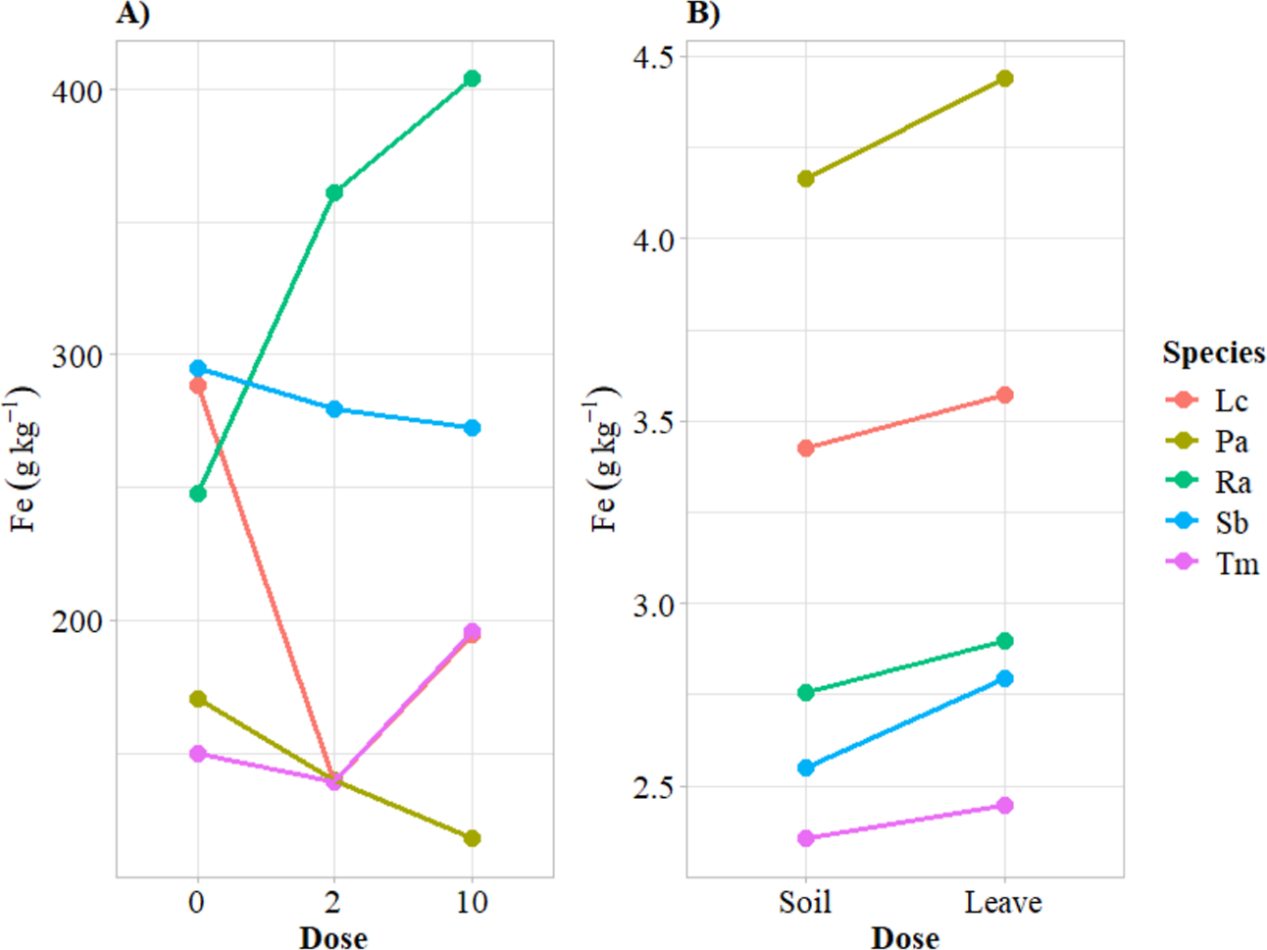
Graphs of interactions for iron. (A) Effects of species-dose interactions on iron (Fe); (B) effects of application method-species interactions on iron (Fe). Species evaluated: Lc = *Lactuca cf. canadensis* L.; Pa = *Pereskia aculeata*; Ra = *Rumex acetose*; Sb = *Stachys byzantina*; Tm = *Tropaeolum majus*.

Iron (Fe) behavior varied significantly across species depending on the Zn dose (Figure 6A). Zinc application decreased Fe concentrations in Lc, Pa and Sb, while it increased Fe concentrations in Ra and Tm. Considering the application method-dose interaction, all species had high concentrations of Fe with foliar Zn application.

### 3.3 Artificial Neural Network Associated with Kohonen Self-Organizing Maps

**Figure 7:**
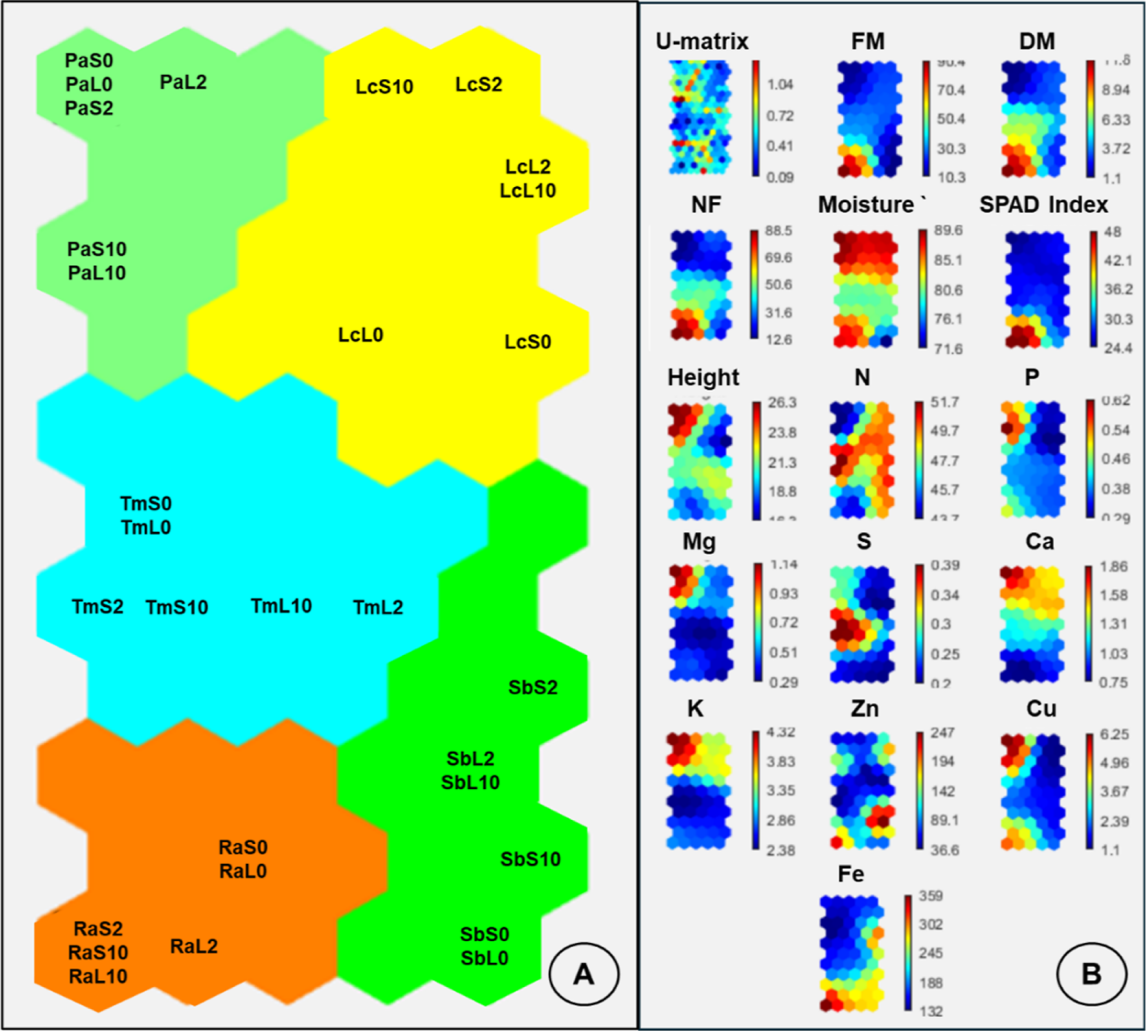
A) Two-dimensional neural cluster map showing the formation of five groups with their respective treatments and UFPs. (B) Component maps and distance matrix (U-matrix) for data relating to fresh mass (FM), dry mass (DM), moisture (Moist), height (He), number of leaves (NL), relative chlorophyll index (SPAD index), hue angle (AH) and the determination of Zn and other minerals (N, P, K, Ca, Mg, S, B, Cu, and Fe).

The results of the Kohonen self-organizing map (KSOM) for the experimental data are shown in Figure 7A,B. Processing the data in the neural network revealed relevant information and trends that may not have been immediately evident from direct data analysis. Furthermore, this approach reduced the dimensionality of data by eliminating redundant information.

In the neural network shown in Figure 7, the correlations between the various input attributes are represented by color-coded values in each variable in the weight vector. Thus, each hexagon in the two-dimensional ANN/KSOM map represents a neuron in which the treatments are grouped according to their similarities.

Based on this assumption, the treatments were divided into five clusters, as shown in the topological map (Figure 7A). Treatments located in neighboring neurons have similar characteristics, highlighting that each species belonged to a specific group. The light green cluster in the top left corner represents only the treatments with Pa. The No cluster, shown in yellow on the right, are treatments with Lc. In the center are the treatments corresponding to Tm, highlighted in blue. Lastly, at the bottom are the remaining two clusters, shown in orange and green, representing Ra and Sb, respectively.

The component maps for each analysis in the Kohonen neural network treatment are presented in Figure 7B. The scale indicates the distance between the neurons, and the variation in the quantitative results is shown by the color gradient of the bars located on the right side of each map, facilitating the identification of which analyses differentiated the samples and which variables were related to the clusters.

Each sample’s position in the neural map (Figure 7A) corresponds to the same position in the component map (Figure 7B), making it possible to identify which variables are responsible for grouping and separating the samples. For instance, the cluster in the top left, shown in green, corresponds to Pa samples, was highly influenced by high values of Moist, K, Mg, Cu, (mainly in applications with 10 kg ha^–1^ of Zn) and Ca, mainly at 0 and 2 kg ha^–1^. Although Mn and Zn values were low, Zn concentrations were high in treatments with 10 kg ha^–1^ of Zn.

The yellow cluster, which included Lc, was separated from the other clusters because it had the lowest values for most variables, except for Moist, N and Ca, which had relatively high values. Zn levels were low, except in the hexagons with leaf samples from plants exposed to 2 and 10 kg ha^–1^, which had high values. Tm, which is shown in blue, is characterized by high AH values. In the lower region of the figure is the orange cluster in orange, representing Ra, which is influenced by the high values of DM, NL, SPAD INDEX, Cu and Fe. These results were observed mainly in treatments with 2 and 10 kg ha^–1^ Zn and foliar applications of 2 kg ha^–1^ Zn.

Lastly, the green cluster in the lower right, representing the treatments applied to Sb, was highly influenced by low values across the analyzed variables compared to the other clusters. The levels of N associated with this species are markedly higher than those associated with other species.

The results of the Kohonen self-organizing map (ANN/KSOM) demonstrate the applicability of this type of ANN to group and describe samples according to their similarity in a visual and intuitive manner and to display relatively substantial amounts of information.

## 4 Discussion

### 4.1 Agronomic Variables

Ra had the highest fresh mass (FM) values regardless of the Zn dose or application method, indicating that this species can easily transport relatively high concentrations of Zn, mainly through the xylem.^22^ In addition, an increase in Zn dose resulted in significant increases in the values of NL, FM and Moist for Ra compared to the other species, revealing a positive correlation between these variables.

The NL per plant is essential for the commercialization of the UFPs evaluated in this research study. NL is generally determined by leaf bundles. The greater the NL, the better the outcome for the producer. In this context, the values of NL for Sb found in this work are greater than those reported by Batista et al. and Silva et al.,^14,30^ who reported 12 to 19 leaves (commercial standard) per plant. For Ra, the results shown here align with those of Silva et al.,^14^ who reported NL values that ranged from 75.98 to 204.08, with an average of 149 leaves per commercial standard. The highest dose of Zn applied foliarly proved more efficient than the other doses, demonstrating the positive impact of the highest dose on the production of these species.

The increase in DM may be related to the nutritional effect of Zn on plants, as this nutrient functions as an enzymatic activator in various metabolic processes, such as cell division and synthesis of proteins, carbohydrates, lipids, and nucleic acids.^20,31,32^

Moisture content is an important index because it can interfere with chemical and biochemical stability, leaf texture and shelf life of species.^15^ Thus, considering the results obtained for Moisture, Zn had a positive effect on the moisture content for Sb and Lc, both of which had lower moisture values when Zn was not applied.

The SPAD index is an indirect indicator of chlorophyll content in leaves and correlates with the nutritional status of plants.^33^ Several studies have shown a positive correlation between the SPAD index and nitrogen (N) content.^34,35^ Such correlation was also observed in this study because, as the foliar application of zinc resulted in important increases in N content in Ra (Figure 3A). These increases are positively correlated with the highest ICR values for this species (Figure 2A). Conversely, the highest dose of Zn significantly decreased the N content in Sb, which, in turn, significantly decreased the SPAD index.

When analyzing the SPAD index alongside the AH variable (Figure 2A,B), it was evident that Pa, Sb and Tm showed similar trends across each variable. In contrast, Lc and Ra showed opposing trends underscoring how much these variables depend on the characteristics of each species, as Zn may be required by many plants for synthetization.^36^ These values demonstrate the positive correlation between these parameters and reinforce the usefulness of noninvasive methods for determining chlorophyll contents.

### 4.2 Nutritive Variables

#### 4.2.1 Macroelements

In vegetables, mineral supply is fundamental to producing photoassimilates and subsequent biomass distribution, especially in leafy crops.^37^ Guo et al.,^38^ studying different doses and application methods of Zn in rice, demonstrated a clear, significant relationship between Zn and N, where the effect of Zn application was positively associated with increased N content. However, in this study, the effects of Zn on N concentration varied by species, with N concentration either increasing or decreasing in response to different doses and application methods. The highest N values were observed in Ra when the highest dose of Zn was applied via foliage. Zn and P have an antagonistic relationship; that is, they interact to promote noncompetitive inhibition of zinc absorption. Torres et al.^39^ explained that P insolubilizes Zn in the xylem, reducing its transport through the plant, while excess Zn inhibits its translocation from the roots to aerial parts of the plant. In this research, only two species (Pa and Ra) showed increased P content due to Zn application, potentially due to their naturally elevated levels of phosphorus in their leaves.^14,30^

The results found for the Mg concentrations may be due to the ability of Zn to competitively inhibit the absorption of Mg, as these elements have similar valences, ionic radii, and degrees of hydration.^40^ The results indicate an antagonistic effect of Zn on Mg in some species when the maximum Zn dose was applied. In Ra, this effect occurred when Zn was applied via soil, whereas in Pa and Lc, it occurred with both application methods. This is a result of the biofortification of these species with Zn; considering that Mg plays an important role in chlorophyll formation and enzymatic activation, it is also necessary for leaves to have the best agronomic and quality characteristics.^41^

When present in plants, sulfur (S) promotes the production of chlorophyll, which is extremely important for protein synthesis.^42^ It also participates in photosynthesis, structural functions, and some redox reactions.^43,44^ Batista et al.^30^ reported that Ra accumulates high levels of S in its leaves; similar results were found in this research when foliar Zn application was used. This may be due to the genetic basis of this species being responsible for the increase in the S content in the leaves, rather than the foliar Zn application.

There were significant differences between species (Figure 4A,B), with a marked reduction in Ca when the highest dose of Zn was applied to Lc and Pa. According to Di Gioia and Rietra et al.,^45,46^ this decrease may be related to competition, as Ca and Zn share membrane transporters. Because calcium regulates plant growth and senescence, root development and biochemical and physiological processes,^41^ its reduction could be detrimental to production.

Except in Ra, K levels decreased with increasing doses of Zn, being even more evident upon foliar application (Figure 4C,D). This reduction occurred because Zn competes with K for the same membrane transport sites.^46^ Foliar application leads to the direct supply of Zn, which represents an advantage of this application method.^45^ As potassium plays a vital role in the growth and development of plants,^44,47^ mitigating its decrease is crucial when biofortification with Zn is carried out.

#### 4.2.2 Microelements

The foliar application of Zn promoted a significant increase in copper (Cu) concentration in Ra and Pa, contrary to other research studies that point the inhibiting effect of zinc on copper.^9^ This inhibitory effect was observed in the other species evaluated (Figure 5B). When Zn was applied via soil, the response varied depending on the species and dose. The direct application of Zn interferes with the absorption of Cu by plants.^48^ Furthermore, when enzymatically bound, Cu participates in various redox reactions in plant metabolism, thus explaining the variation in its concentration in some species.

The results in Figure 6 indicate a reduction in iron (Fe) content in Lc, Pa and Sb. This reduction in the edible parts of plants may be due to an inversely proportional correlation between Zn and Fe in leaves, given the similarity in their atomic radii, causing them to compete for absorption sites.^41^ da Silva Sousa et al.,^49^ who studied Zn biofortification in vegetables, also reported that increasing Zn doses reduced Fe content in edible parts of vegetables.

In tropical Oxisols typical of Brazil, such as the one used in this research, zinc uptake is generally low, first due to the availability of this nutrient in native soils,^50^ where Zn values are 0.8 mg dm^–3^ (Table 1), classified as low according to different authors;^51,52^ and second, due to the low OM contents in these soils (Table 1). de Morais et al. and Dhaliwal et al.^50,53^ stated that the organic matter of the soil affects the dynamics and availability of metallic micronutrients through several mechanisms, such as adsorption on the surface of organic functional groups or the formation of organic complexes with greater solubility and mobility. de Morais et al.^50^ demonstrated that lower OM content in the soil correlates to reduced concentrations of microelements in the soil solution. Thus, to prevent the problem of Zn fixation, foliar Zn application has the added advantage of reducing the distance Zn must travel; with foliar applications, Zn has a shorter distance to move within the plant.^54^

Therefore, the foliar application of Zn has proven to be an important management practice for improving the nutritional quality of UFPs. This method not only improves morphological aspects of the plants, such as He, FM and DM, but also aligns with previous research,^55,56^ confirming the claims of Januszkiewicz et al.,^57^ which indicated the importance of increasing the concentrations of essential components for both human and animal nutrition in plant foods as the main objective of foliar fertilization. However, despite the greater efficiency of foliar application, nutrient absorption is dependent on the thickness of the upper and lower leaf cuticle surfaces of each species as well as the number of pores and the distribution of trichomes and stomata on the leaf surface.^51^ These variables may explain the variation in leaf Zn gain depending on the species.

In general, most plants require foliar Zn concentrations greater than 15–30 mg kg^–1^ of dry weight to maintain vital and metabolic functions. However, in nonhyperaccumulative species, foliar Zn concentrations above 100–700 mg kg^–1^ of dry weight are toxic, causing several negative effects, but mainly a reduction in growth and suppression of production.^23^ Therefore, Ra can be considered a Zn bioaccumulator, as they were not associated with a reduction in growth or leaf production, even at values greater than 1000 g kg^–1^, which were observed when foliar Zn was applied.

## 5 Conclusion

The species evaluated responded differently to the applied Zn doses, which influenced both nutrient absorption and the development of UFPs. The application of Zn promoted positive effects on agronomic parameters, including dry mass and number of leaves. Foliar Zn application proved to be more efficient than soil Zn application, especially to nutritional parameters.

*R. acetosa* was the species most responsive to Zn applications, regardless of the application method. This species presented notable values for productive indicators, such as fresh mass, dry mass and number of leaves, in addition to presenting the highest Zn concentrations when the highest dose was administered via foliar application.

Through biofortification with Zn, *R. acetosa* has the potential to be a UFP capable of reducing nutritional deficiencies of Zn within the population when incorporated into daily diets.
